# An Integrated CT and MRI Imaging Model to Differentiate between Adrenal Adenomas and Pheochromocytomas

**DOI:** 10.3390/cancers15143736

**Published:** 2023-07-23

**Authors:** Marta Araujo-Castro, Iñigo García Sanz, César Mínguez Ojeda, María Calatayud, Felicia A. Hanzu, Mireia Mora, Almudena Vicente Delgado, Concepción Blanco Carrera, Paz de Miguel Novoa, María del Carmen López García, Laura Manjón-Miguélez, Pablo Rodríguez de Vera Gómez, María del Castillo Tous, Rebeca Barahona San Millán, Mónica Recansens, Mariana Tomé Fernández-Ladreda, Nuria Valdés, Paola Gracia Gimeno, Cristina Robles Lazaro, Theodora Michalopoulou, Victoria Gómez Dos Santos, Cristina Alvarez-Escola, Rogelio García Centeno, Cristina Lamas, Aura Herrera-Martínez

**Affiliations:** 1 Endocrinology & Nutrition Department, Hospital Universitario Ramón y Cajal, Instituto de Investigación Biomédica Ramón y Cajal (IRYCIS), 28034 Madrid, Spain; 2 Medicine Departmen, University of Alcalá, 28801 Madrid, Spain; 3 General & Digestive Surgery Department, Hospital Universitario de La Princesa, 28006 Madrid, Spain; 4 Urology Department, Hospital Universitario Ramón y Cajal, 28034 Madrid, Spainvgomezd69@gmail.com (V.G.D.S.); 5 Endocrinology & Nutrition Department, Hospital Universitario Doce de Octubre, 28041 Madrid, Spain; 6 Endocrinology & Nutrition Department, Hospital Clinic, 08036 Barcelona, Spain; 7 Endocrinology & Nutrition Department, Hospital Universitario de Toledo, 45007 Toledo, Spain; almudena.vicente@m3d.net; 8 Endocrinology & Nutrition Department, Hospital Universitario Príncipe de Asturias, 28805 Madrid, Spain; conchablanco.carrera@gmail.com; 9 Endocrinology & Nutrition Department, Hospital Clínico San Carlos, 28040 Madrid, Spain; pazdemiguelnovoa@gmail.com; 10 Endocrinology & Nutrition Department, Hospital Universitario de Albacete, 02008 Albacete, Spain; maricarmenlopez1b@gmail.com (M.d.C.L.G.); clamaso@sescam.jccm.es (C.L.); 11 Endocrinology & Nutrition Department, Hospital Universitario Central de Asturias, Instituto de Investigación Sanitaria del Principado de Asturias (ISPA), 33011 Oviedo, Spain; lauramanjonmiguelez@gmail.com; 12 Endocrinology & Nutrition Department, Hospital Universitario Virgen de la Macarena, 41009 Sevilla, Spain; pablordevera@gmail.com (P.R.d.V.G.); mariatous2@gmail.com (M.d.C.T.); 13 Endocrinology & Nutrition Department, Institut Català de la Salut Girona, 17001 Girona, Spain; rbarahona.girona.ics@gencat.cat (R.B.S.M.); mrecasens.girona.ics@gencat.cat (M.R.); 14 Endocrinology & Nutrition Department, Hospital Universitario de Puerto Real, 11510 Cádiz, Spain; marianatomefl@yahoo.es; 15 Endocrinology & Nutrition Department, Hospital Universitario de Cabueñes, 33394 Asturias, Spain; nvalga@gmail.com; 16 Endocrinology & Nutrition Department, Hospital Royo Villanova, 50015 Zaragoza, Spain; paogracia@gmail.com; 17 Endocrinology & Nutrition Department, Hospital Universitario de Salamanca, 37007 Salamanca, Spain; crisrlz@hotmail.com; 18 Department of Endocrinology and Nutrition, Joan XXIII University Hospital, 43005 Tarragona, Spain; tmichalopoulou.hj23.ics@gencat.cat; 19 Endocrinology & Nutrition Department, Hospital Universitario La Paz, 28046 Madrid, Spain; escola.cristina@gmail.com; 20 Endocrinology & Nutrition Department, Hospital Universitario Gregorio Marañón, 28029 Madrid, Spain; roges82@msn.com; 21 Department of Endocrinology and Nutrition, Reina Sofía Hospital, 31500 Córdoba, Spain; aurita.dhm@gmail.com

**Keywords:** adrenal adenoma, atypical adrenal adenoma, adrenal tumor, high lipid content, pheochromocytoma

## Abstract

**Simple Summary:**

The information obtained in the CT and MRI allows adequate differentiation of adrenal adenomas and pheochromocytomas (PHEOs) with a high diagnostic accuracy. Our study confirms that our predictive model combining tumor size and lipid content has high re-liability for the prediction of PHEO when atypical adrenal lesions are excluded. However, for atypical adrenal lesions with >10 HU in an unenhanced CT scan, MRI information is necessary for a proper exclusion of the PHEO diagnosis. We observed that in the whole cohort (including atypical adenomas), when MRI information was included in the model, the diagnostic accuracy increased to up to 85% when the variables tumor size, high lipid content in an unenhanced CT scan, and hyperintensity in the T2 sequence in MRI were included.

**Abstract:**

Purpose: to perform an external validation of our predictive model to rule out pheochromocytoma (PHEO) based on unenhanced CT in a cohort of patients with PHEOs and adenomas who underwent adrenalectomy. Methods: The predictive model was previously developed in a retrospective cohort of 1131 patients presenting with adrenal lesions. In the present study, we performed an external validation of the model in another cohort of 214 patients with available histopathological results. Results: For the external validation, 115 patients with PHEOs and 99 with adenomas were included. Our previously described predictive model combining the variables of high lipid content and tumor size in unenhanced CT (AUC-ROC: 0.961) had a lower diagnostic accuracy in our current study population for the prediction of PHEO (AUC: 0.750). However, when we excluded atypical adenomas (with Hounsfield units (HU) > 10, n = 39), the diagnostic accuracy increased to 87.4%. In addition, in the whole cohort (including atypical adenomas), when MRI information was included in the model, the diagnostic accuracy increased to up to 85% when the variables tumor size, high lipid content in an unenhanced CT scan, and hyperintensity in the T2 sequence in MRI were included. The probability of PHEO was <0.3% for adrenal lesions <20 mm with >10 HU and without hyperintensity in T2. Conclusion: Our study confirms that our predictive model combining tumor size and lipid content has high reliability for the prediction of PHEO when atypical adrenal lesions are excluded. However, for atypical adrenal lesions with >10 HU in an unenhanced CT scan, MRI information is necessary for a proper exclusion of the PHEO diagnosis.

## 1. Introduction

Adrenal incidentalomas are one of the most frequent reasons for consultation in endocrinology. They are present in 4% of the general population and in up to 10% of elderly patients [1]. The two main clinical issues to be determined in this setting are the risk of malignancy and the hormonal activity of these lesions [2]. The radiological features of the adrenal lesion usually allow easy discrimination between malignant and benign tumors [3]. However, the functional evaluation of an adrenal incidentaloma is generally complex, requiring evaluation of both hypercortisolism and catecholamine hypersecretion in all patients with a new diagnosis of an adrenal lesion [3].

Autonomous cortisol secretion is a very common condition in patients with adrenal incidentalomas, reaching a prevalence of up to 40% [4]. In addition, the 1 mg dexamethasone suppression test is an inexpensive and easy-to-perform test. Nevertheless, pheochromocytoma (PHEO) represents less than 5% of all adrenal incidentalomas [5]. Moreover, the measurement of urine or plasmatic fractionated metanephrines is expensive, cumbersome, and time-consuming and may be affected by several drugs and diet components, often leading to falsely elevated results [6]. Furthermore, 50% of PHEO patients have only mild elevations in the biochemical markers [7]. However, the radiological features of the adrenal lesion have been reported to be very precise in determining whether the adrenal lesion is a PHEO or not [8,9,10,11]. In this regard, we previously developed a predictive model of PHEO in a cohort study of 1131 patients presenting with adrenal lesions, including 163 subjects with histological confirmation of PHEO and 968 patients showing no clinical suspicion of PHEO in whom plasma and/or urinary metanephrines and/or catecholamines were within reference ranges. We found that the combination of high lipid content and tumor size in an unenhanced CT scan had a high diagnostic accuracy for the diagnosis of PHEO (AUC-ROC: 0.961). The probability of having a PHEO was 0.1% for adrenal lesions smaller than 20 mm showing high lipid content in unenhanced CT (<10 HU). Thus, we have suggested avoiding biochemical screening for PHEO in patients with adrenal lesions smaller than 20 mm showing high lipid content in the unenhanced CT scan. However, we did not perform an external validation of the model to confirm the usefulness of this predictive model in other study populations.

Considering this background, the aim of our study was to perform an external validation of our previously described predictive model of PHEO (based on the results of unenhanced CT) in a large cohort of patients with a histologically confirmed diagnosis of PHEO and adrenocortical adenoma, including patients with atypical adrenal adenomas with low lipid content (Hounsfield units (HU) > 10).

## 2. Materials and Methods

This retrospective multicenter study was approved by the Hospital Universitario Ramón y Cajal Ethics Committee, and a waiver of informed consent was granted.

### 2.1. Study Population for the External Validation

We included 214 patients with adrenal lesions evaluated at 13 tertiary academic hospitals between 2001 and 2022 whose imaging (CT and/or MRI) data were available.

**Figure 1:**
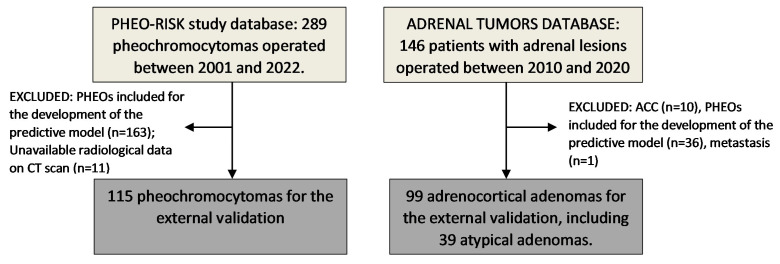
Study population. ACC: adrenocortical carcinoma; CT: computed tomography; PHEO: pheochromocytoma.

Two groups of patients with available histopathological results were included: (i) patients with histological confirmation of pheochromocytoma (PHEO group), not previously included in the cohort study for the development of the predictive model, and (ii) patients with histological confirmation of adrenocortical adenoma (ADENOMA group). This second group was extracted from the ADRENAL-PATHOLOGY database of the Ramón y Cajal Hospital [12]. Patients in the first group were selected from the PHEO-RISK study database, as previously described [13]. Patients of both groups were identified through a systematic electronic search in the Pathology, Endocrinology, Biochemistry, and/or Admission department files of the different hospitals (Figure 1).

### 2.2. Clinical, Hormonal, and Radiological Data

Medical records were reviewed retrospectively to extract demographic information such as age and sex, medical history of comorbidities at diagnosis, and physical examination variables including body mass index (BMI) and systolic and diastolic blood pressure. The hormonal study consisted in the evaluation of catecholamine excess by the measurement of urinary or plasma-free metanephrines and/or urinary catecholamines.

All the patients included in the study had the available information about radiological data on unenhanced CT scans at diagnosis. In addition, 92 patients had the available information about MRI radiological parameters. Different equipment and image acquisition protocols were used throughout the study periods at different institutions. The following information was registered in the unenhanced CT scan: tumor size (largest reported diameter), uni- or bilaterality, lipid content measured in the unenhanced phase of the CT scan, presence of calcifications or necrosis, and HU. For bilateral adrenal lesions, the size of the largest adenoma was included in the analyses. Adrenal tumors were considered rich in lipid content when attenuation was low (<10 HU) in a CT scan performed without intravenous contrast [2]. If attenuation was >10 HU, they were classified as atypical adenomas. MRI information included: size and chemical shift imaging—which allows the detection of intracellular lipid that is contained in the most frequent adrenal lesions (adenomas) with loss of signal in the “out of phase” sequence—and evaluation of hyperintensity in the T2 sequence.

### 2.3. Statistical Analysis

Continuous variables were expressed as mean ± standard deviation, and categorical variables were described as proportions. For variables with some missing data, we have indicated the number of patients with available results in brackets in the different tables. Shapiro Wilk’s test was used to assess the normality of continuous variables. Student’s *t* test was used for the comparison of continuous variables and the χ2 test for the comparison of proportions among the groups of patients. The predictive model was developed using a multivariate logistic regression model. A two-tailed *p* value < 0.05 was considered as statistically significant in all analyses.

The selection of variables for the model was based on the results of the univariate logistic regression model to predict PHEO, and only variables with less than 30% of missing results were considered to enter in the predictive model. The estimation of all possible equations was used to select the model with the best diagnostic accuracy (lower Akaike index (AIC) and maximum C Harrell index). The receiver operating characteristic curve (ROC) analysis was used as a measure of the diagnostic accuracy of the different selected predictive models and to identify the cutoff values with the best combination of sensitivity and specificity. All statistical data analyses were performed with STATA 15.0 (StataCorp LLC, College Station, TX, USA).

## 3. Results

### 3.1. Predictive Model of PHEO

**Figure 2:**
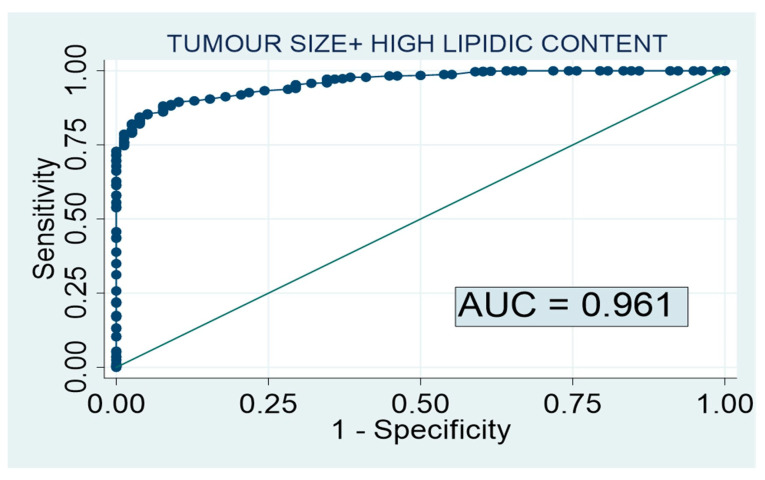
Predictive model of PHEO, previously described in a cohort of 1131 patients [14]. AUC 0.961 [0.946–0.976]; Based on optimal threshold: sensitivity 88.1%; Specificity 92.3%.

As we have previously described [13], 163 patients with PHEO and 968 subjects with non-PHEO lesions were employed for the development of the PHEO predictive model. The combination of tumor size and high lipid content in the unenhanced CT scan achieved a diagnostic accuracy of 96.1% for the diagnosis of non-PHEO lesions (Figure 2). Based on this predictive model, the probability of having a PHEO in an adrenal lesion smaller than 20 mm with high lipid content in the CT scan was 0.1%.

### 3.2. Baseline Characteristics of the Patients Included for the External Validation

For the external validation, 115 patients with PHEOs and 99 with adenomas were enrolled in the study. Of the 99 patients with adenomas, 30 had primary hyperaldosteronism, 18 overt Cushing syndrome, 9 autonomous cortisol secretion, and 42 non-functioning adrenal adenomas (in whom surgery was indicated due to a tumor size > 4 cm and/or the presence of atypical radiological features). In the group with adenomas, a total of 25 patients had atypical adrenal adenomas, with HU > 10 and a tumor size > 40 mm; 14 had HU > 10 but with a tumor size < 40 mm; and 11 had typical benign features with a tumor size > 40 mm. The other cases were operated due to functionality.

The baseline clinical, hormonal, and radiological features of the patients with PHEOs and adenomas are described in Table 1. The patients with PHEOs were younger and had cardiovascular disease and obesity more often than patients with adrenal adenomas (Table 1). In addition, tumor characteristics were significantly different between the groups, including a larger tumor size, higher HU in the unenhanced CT scan, and a greater prevalence of necrosis and hyperintensity in the T2 sequence in MRI in PHEOs compared with adenomas. On the other hand, loss of signal in the “out of phase” sequence of MRI was common in adrenal adenomas, but it was present only in two patients of the PHEO group. The two cases with PHEOs who had tumors with loss of signal in the “out of phase” sequence of MRI presented other radiological features not typical of adenomas, including >30 HU and a tumor size > 55 mm in both cases. However, these two cases did not present hyperintensity in the T2 MRI sequence either.

### 3.3. External Validation of Our Previous Predictive Model and New Proposed Models

When we applied our previously reported predictive model that combined tumor size and lipid content in an unenhanced CT scan [13] to our patients with available histological results of the adrenal lesion, the AUC of the model was quite low compared with our previously reported result (AUC: 0.750 [95% CI: 0.677–0.814]). Despite the lower diagnostic accuracy of the model in our current population, the probability of having a PHEO was lower than 2% for adrenal lesions of <10 HU and a tumor size < 20 mm (Table 2). When we excluded atypical adenomas (with HU > 10, n = 39) from the external validation cohort, the AUC of the model reached a diagnostic accuracy of 87.4% [95% CI: 0.800–0.925].

**Figure 3:**
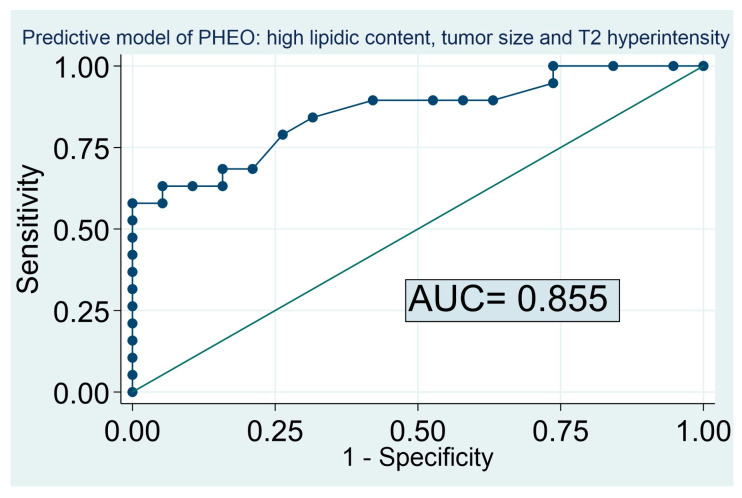
Predictive model of PHEO in adrenal lesions (including atypical adenomas). AUC 0.855 [CI 0.687–0.940]. Based on optimal threshold: sensitivity 79%; Specificity 74%; and based on the threshold for maximum efficiency: Sensitivity of 57.9% and specificity of 100.0%.

The inclusion of the radiological information of the MRI in the model allowed an increase in the diagnostic accuracy of the predictive model in the global cohort (including atypical adenomas), reaching an accuracy of 85% for the prediction of PHEO when the variables tumor size, high lipid content in an unenhanced CT scan, and hyperintensity in the T2 sequence in the MRI were included (AUC: 0.855, 95% CI: 0.687–0.940) (Figure 3). Based on this predictive model, the probability of PHEO was lower than 0.3% for adrenal lesions with >10 HU, with a tumor size < 20 mm and without hyperintensity in the T2 sequence. On the other hand, the probability of PHEO exceeded 90% for adrenal lesions with low lipid content, a tumor size > 20 mm, and hyperintensity in the T2 sequence (Table 3). In our cohort, no patient with PHEO had a tumor with high lipid content and an absence of hyperintensity in T2 (pretest probability of PHEO of 0%). The combination of the variables tumor size, high lipid content in an unenhanced CT scan, and loss of signal in the “out of phase” sequence and hyperintensity in the T2 sequence in MRI increased the accuracy of the model to up to 89% [95% CI: 0.750 to 0.980].

## 4. Discussion

The predictive model based on tumor size and lipid content in an unenhanced CT scan had a good diagnostic accuracy for the prediction of PHEO when atypical adenomas were excluded from the model. However, for the prediction of PHEO in adrenal lesions with low lipid content, in addition to tumor size it was necessary to have information about hyperintensity in the T2 MRI sequence to achieve a reliable exclusion of the PHEO diagnosis.

When we tested the diagnostic accuracy of our previously reported PHEO predictive model in our cohort of operated patients, the diagnostic accuracy was quite low (75%) compared with our previously reported result (AUC of 0.961 when non-operated adrenal lesions with normal metanephrine levels were included as the control group, and an AUC of 0.737 when we used a cohort of patients with operated adrenal adenomas). However, when we included in the control group of non-PHEO patients only those cases with typical adenomas, the diagnostic accuracy reached 87%. These findings are probably related to the fact that the patients with adrenocortical adenomas who underwent adrenalectomy may have had larger tumors with atypical features or a significant enlargement during follow-up. In this regard, it is important to consider that approximately 20% of adrenal adenomas are classified as atypical [14,15]. In contrast with the literature, in our series approximately 40% of the patients had atypical adenomas. Atypical adenomas do not display the typical intracellular microscopical fatty content, and for this reason they are also called lipid-poor adrenal adenomas. It is not surprising that the prevalence of atypical adenomas was higher in our study than in the study reported by other authors [16], since the rate of malignant and atypical adrenal lesions is usually higher in surgical series than in clinical studies. The predictive model of PHEO based on tumor size and high lipid content had a diagnostic accuracy higher than 85% when atypical adenomas were excluded. These results support our previous recommendation of avoiding biochemical screening for PHEO in patients with adrenal lesions smaller than 20mm showing high lipid content in the CT scan if there are no typical signs and symptoms of pheochromocytoma [13]. It is known that the most challenging task is to differentiate between atypical adrenal adenomas and PHEOs. Previous studies have proposed different methods for their differentiation [17,18,19]. For example, a recent study proposed a formula combining cystic degeneration, attenuation values in unenhanced CT, and the peak value of enhancement in the arterial and venous phases for the differentiation of both conditions, reaching an accuracy of 95% in the training cohort and 91% in the external validation population [17]. Nagayama et al. [18] reported that a relative enhancement ratio of the venous phase to unenhanced CT of ≥210% allows an accurate differentiation between lipid-poor adenomas and PHEOs. Yong-Yu An et al. [19] proposed a predictive model combining a lesion size of >29 mm, an arterial-phase attenuation of >81 HU, a venous-phase attenuation of >97 HU, an enhancement ratio in the venous phase of ≤1.5, and the presence of cystic degeneration. This diagnostic scoring model yielded an AUC of 0.911. However, this model includes several radiological parameters that are not always described in radiological reports, making its implementation in clinical practice more complicated than our proposed predictive model.

When evaluated using MRI, PHEOs demonstrate a hypointense signal on T1-weighted images and a hyperintense signal on T2-weighted (T2W) images. Classically, PHEOs were considered to have a characteristic T2W hyperintensity, known as the “lightbulb bright” appearance [20]. However, this hyperintensity is not present in approximately 35% of PHEOs [21], reflecting the limitations of this technique when used alone. Remarkably, in our cohort, MRI provided additional information when T2W hyperintensity was included in the model. Similarly, Schieda et al. [22] compared quantitative MRI and washout CT analysis in the differential diagnosis of PHEO and adenoma. They observed that PHEOs had a lower chemical shift signal intensity index, higher adrenal-to-spleen signal intensity ratio, and higher T2-weighted signal intensity ratio; in this context, they did not observe statistically significant differences in contrast-enhanced MRI AUCs. Despite this observation, when the T2W intensity ratio was analyzed using values greater than 3.8, the AUC reached 0.91 and was diagnostic of nearly two-thirds of PHEOs. Regarding the chemical shift imaging in MRI, it is considered a reliable tool for the differentiation of benign and malignant adrenal mass [3]. However, in our study up to 7% (2 out of 28) of the PHEO cases had a loss of signal in the “out of phase” sequence of the MRI, indicating a low specificity for the differential diagnosis of PHEO vs. adenoma. The higher frequency of areas of fatty degeneration in patients with PHEOs may explain the signal drop on chemical shift imaging in some cases [23].

Some recent studies have described the usefulness of the use of texture analysis or machine learning of radiomic features for the diagnosis of PHEO [24,25,26]; however, these results have some limitations related to software availability and the lack of external validation. Recently, a study reported the usefulness of a T2W adrenal (qualitative and quantitative) calculator that combines T2W signal intensity ratio and entropy to differentiate lipid-poor adrenal adenomas from metastases [27]. In this study, three blinded radiologists measured the T2W signal intensity (SI) ratio (SInodule/SIpsoas muscle), T2-weighted histogram features, and chemical shift SI index, and reported an AUC for the T2W SI ratio and T2W entropy of 0.76 and 0.94, respectively. Additionally, they performed a logistic regression model combining the T2W SI ratio with T2W entropy, resulting in an AUC of 0.95. Based on this model, Gerson et al. [28] used a T2W MRI calculator to differentiate PHEO from lipid-poor adrenal adenoma. Specifically, this study showed that subjective T2W heterogeneity and T2W entropy (using a first-order texture analysis) were both significantly higher in PHEOs than in atypical adenomas; when they imputed the T2W signal intensity ratio and T2W entropy values into the quantitative T2W MRI calculator, it differentiated PHEOs from adenomas with high sensitivity, specificity, and accuracy (93–100%, 87–96%, and 94–96%, respectively). As in our study, this model included lesions of different sizes, but it still requires an external validation, and, in contrast to our cohort, only the PHEOs were confirmed by histology. These studies reveal the relevant role of T2W MRI images in the diagnosis of adrenal lesions and reflect the importance of non-invasive diagnostic techniques, based on imaging, for the diagnosis of adrenal tumors. As in our cohort, imaging-based predictive models could represent a low-cost alternative to nuclear medicine techniques in selected cases, including screening patients with an underlying genetic predisposition to PHEO. Other important studies in the field of the machine learning approach for adrenal lesion characterization include the study by Romeo V. [29] that reported a diagnostic accuracy of 80% using this approach and a series by Stanzione A. [30] reporting a cross-validation accuracy of 0.94 on the train and 0.91 on the test sets with the machine learning method.

We are aware that our study has some limitations. First, it has a retrospective design, which is prone to selection bias and missing data. Moreover, discrepancies in the measurements and radiological analyses performed by the different radiologists across the different centers might have occurred. However, despite these limitations, our research is of great value for clinical practice because we have studied a large cohort of operated adrenal lesions and found that it is possible to avoid the biochemical screening of PHEO in all patients with tumors with high lipid content and an absence of hyperintensity in T2, since the probability of PHEO is as low as 0%.

## 5. Conclusions

Our study confirms that our predictive model combining tumor size and lipid content has high reliability for the prediction of PHEO when atypical adrenal lesions are excluded. However, for atypical adrenal lesions with >10 HU in an unenhanced CT scan, MRI information is necessary for a proper exclusion of the PHEO diagnosis. Based on our model combining CT and MRI information, the probability of PHEO is lower than 0.3% for adrenal lesions with >10 HU with a tumor size < 20 mm and without hyperintensity in T2. On the other hand, the probability of PHEO exceeded 90% for adrenal lesions with low lipid content, a tumor size > 20 mm, and hyperintensity in the T2 sequence. Thus, the hormonal evaluation with metanephrine and/or catecholamine is not necessary in the first scenario and is mandatory in the group of patients with a high pretest probability.

## Figures and Tables

**Table 1:** Baseline characteristics of patients with PHEOs and adenomas.

	PHEO (n = 115)	ADENOMA (n = 99)	*p* Value
**Clinical and Hormonal Data**			
Age (years)	49.7 ± 18.06	55.9 ± 11.20	0.004
Female sex	47.0% (n = 54)	39.4% (n = 39)	0.266
Hypertension	50.4% (n = 58)	63.6% (n = 63)	0.052
Type 2 diabetes	25.2% (n = 29)	30.3% (n = 30)	0.406
Dyslipidemia	29.6% (n = 32)	28.3% (n = 28)	0.831
Cardiovascular events	10.4% (n = 12)	3.3% (n = 3)	0.034
Cerebrovascular events	5.4% (n = 6)	3.1% (n = 3)	0.404
Obesity	19.1% (n = 22)	8.1% (n = 8)	0.020
Systolic blood pressure (mmHg)	137.7 ± 22.20	137.9 ± 11.91	0.952
Diastolic blood pressure (mmHg)	81.2 ± 14.65	79.8 ± 10.09	0.425
Body mass index (kg/m^2^)	26.4 ± 4.87	26.4 ± 3.17	0.973
Urinary metanephrine (mcg/24 h)	2521.4 ± 5405.26	339.2 ± 133.11	<0.001
**Unenhanced CT scan**			
Tumor size (mm)	52.0 ± 31.80	37.0 ± 19.47	<0.001
Tumor size > 40 mm	61.7% (n = 66/107)	47.5% (n = 47/99)	0.041
Hounsfield Units (n = 86)	45.3 ± 27.3	29.9 ± 31.89	0.035
Hounsfield Units >10 (n = 86)	97.1% (n = 34/35)	65.0% (n = 39/60)	<0.001
Bilaterality	5.1% (n = 5/99)	6.1% (n = 6/99)	0.756
Necrosis	20.2% (n = 18/89)	2.2% (n = 2/90)	<0.001
Calcifications	6.6% (n = 5/76)	16.7% (n = 15/90)	0.047
High lipid content	1.3% (n = 1/75)	26.7% (n = 24/90)	<0.001
**MRI evaluation**			
Loss of signal in the “out of phase” sequence	7.1% (n = 2/28)	72.7% (n = 24/33)	<0.001
Hyperintensity in T2 sequence	64.9% (n = 24/37)	15.2% (n = 5/33)	<0.001

CT: computerized tomography; MRI: magnetic resonance imaging.

**Table 2:** Probability of PHEO based on tumor size and lipid content in unenhanced CT scan in patients with adrenal lesions, including atypical adenomas.

	Tumour Size	<10 mm	10–20 mm	21–30 mm	31–40 mm	41–50 mm	>50 mm
Lipid Content							
High		1.1%	1.6%	2.4%	3.5%	5.1%	7.3%
Low		23.1%	30.7%	39.7%	49.4%	59.1%	68.2%

**Table 3:** Probability of PHEO based on tumor size and hyperintensity in T2 in MRI in adrenal lesions with low lipid content in CT scan.

		**HYPERINTENSITY IN T2 MRI SEQUENCE**					
	**Tumour Size**	**<10 mm**	**10–20 mm**	**21–30 mm**	**31–40 mm**	**41–50 mm**	**>50 mm**
**Lipid Content**							
**Low**		37.2%	72.4%	92.1%	98.1%	99.6%	99.9%
		**ABSCENSE OF HYPERINTENSITY IN T2 MRI SEQUENCE**					
	**Tumour Size**	**<10 mm**	**10–20 mm**	**21–30 mm**	**31–40 mm**	**41–50 mm**	**>50 mm**
**Lipid Content**							
**Low**		0.1%	0.3%	1.2%	5.2%	19.5%	51.8%

## Data Availability

All data generated or analyzed during this study are included in this article. Further enquiries can be directed to the corresponding author.
